# Clinical progression and outcomes of 260 patients with severe COVID-19: an observational study

**DOI:** 10.1038/s41598-021-82943-5

**Published:** 2021-02-04

**Authors:** Junjuan Wang, Xulei Zheng, Jianbin Chen

**Affiliations:** 1 Department of Neurosurgery, Tongji Hospital, Tongji Medical College, Huazhong University of Science and Technology, Wuhan, 430030 Hubei China; 2 School of Basic Medical Sciences and Forensic Medicine, Hangzhou Medical College, Hangzhou, Zhejiang China; 3 Department of Obstetrics and Gynecology, Tongji Hospital, Tongji Medical College, Huazhong University of Science and Technology, Wuhan, Hubei China

**Keywords:** Diseases, Infectious diseases, Viral infection

## Abstract

This paper presents the results of an observational, prospective study of the clinical progression and outcomes of patients with severe COVID-19. Overall, 260 patients with severe COVID-19 were included. The median age of the patients was 61 years (IQR 42.0–73.0), and 119 (45.8%) patients had one or more medical comorbidities. The median time from initial onset of symptoms to hospital admission was 8 days (IQR 6.0–11.0). Varying degrees of abnormalities in blood biochemical results were detected in most patients. All patients received supportive therapy and antiviral treatment. All patients were administered empirical antibiotic treatment with a median time of 5 days (IQR 3–7). Mechanical ventilation was required in accordance with respiratory conditions. At the data cutoff, 183 (70.4%) patients had been discharged, and 17 (6.5%) patients had been transferred to the intensive care unit (ICU). Twenty-five (9.6%) patients had died, and 35 (13.5%) patients were still in the hospital. During follow-up, 7 patients with fever were negative for SARS-Cov-2 antigens upon retest. The implications of the results are discussed for clinical features and the management of patients with severe COVID-19.

## Introduction

In December 2019, the novel coronavirus caused a global pandemic of viral pneumonia termed coronavirus disease 2019 (COVID-19)^1–3^. By Jan 7, 2020, deep sequencing analysis from lower respiratory tract samples of these patients indicated a novel coronavirus with severe acute respiratory syndrome coronavirus 2 (SARS-CoV-2; previously known as 2019-nCoV)^4,5^. Coronaviruses are enveloped nonsegmented positive-sense RNA viruses belonging to the family Coronaviridae and the order Nidovirales are broadly distributed in humans and other mammals^4,5^. The initial coronavirus infection was believed to have been transmitted from animals, and it was confirmed that secondary affected patients had been infected with the virus through human to human transmission^1,2,4–6^. The rapid increased cases has sustained. By early January, 2021, there were more than 212 countries with COVID-19 epidemic. And there were in total more than 85.7 million cases with confirmed COVID-19, 23.6 million confirmed COVID-19 at present, and more than 1.85 million deaths worldwide.

The clinical classification of COVID-19 ranges from asymptomatic, to mild, severe, or critical^6–8^. Although most COVID-19 patients are mild, there are also a few severe and critical patients^6–8^. The rapid increased cases in highly contagious cases made for an extreme shortage of local medical resources and serious inadequacies of the isolation ward. The Chinese government and medical profession took a prompt response to the COVID-19 epidemic. All confirmed cases with of SARS-CoV-2 infection were centrally isolated and treated in designated hospitals. According to the severity of illness, all cases were classified and admitted to cargo hospitals (asymptomatic and mild), general wards (severe) or intensive care units ([ICU], critical).

As the number of confirmed cases increases, the clinical features and outcomes of severe patients, which account for the second largest number^7^, remain unknown and require further exploration. We performed a comprehensive exploration of the clinical features and short-term outcomes of patients with severe COVID-19 to provide insight into treatment and post-discharge management around the world.

## Results

Overall, 260 severe patients with COVID-19 were admitted to the isolation ward of our hospital (Table 1). The demographics and baseline characteristics are summarized in Table 1. In total, the median age of the patients was 61 years (IQR 42.0–73.0; mean = 57.2 ± 14.9 years), with 148 (56.9%) patients aged 50–82 years, and 98 (37.7%) patients aged 30–49 years. The male to female ratio was approximately 1:1 (132:128). Thirty-nine (15%) patients had a history of exposure to the Huanan seafood market. The remaining 85% patients had no known history of exposure to the market, but all lived in Wuhan 14 days before the onset of illness. Twenty-eight (10.8%) patients were family cluster members, who came from 10 families. Three (1.2%) patients were pregnant and 1 (0.4%) patient was postpartum. Eight (3.1%) patients were medical staff, and 119 (45.8%) patients had a history of one or more coexisting chronic illnesses, including cardiovascular disease in 56 (21.5%) patients, cerebrovascular disease in 42 (16.2%) patients, digestive system disease in 38 (14.6%) patients, diabetes in 30 (11.5%) patients, chronic renal insufficiency in 19 (7.3%) patients, respiratory system disease in 14 (5.4%) patients, nervous system disease in 6 (2.3%) patients, malignant tumor in 5 (1.9%) patients and rheumatism and connective tissue disease in 3 (1.2%) patients. The number of comorbidities presented as follows: 54.2% (141) of patients presented with no comorbidities, 23.8% (62) with 1 comorbidity, 10.4% (27) with 2 comorbidities, 6.2% (16) with 3 comorbidities, 2.3% (6) with 4 comorbidities, and 3.1% (8) with 5 comorbidities.

**Table 1:** Clinical characteristics of patients with COVID-2019 on admission.

	Patients (n = 260)
**Age, years**	
Mean (SD), range	56.2 ± 14.9, 14–81
14–18	2 (0.8%)
18–29	12 (4.6%)
30–39	48 (18.5%)
40–49	50 (19.2%)
50–59	57 (21.9%)
60–69	53 (20.4%)
70–79	24 (9.2%)
80–82	14 (5.4%)
**Sex**	
Male	132 (50.8%)
Female	128 (49.2%)
**Exposure to huanan seafood market**	
Yes	39 (15%)
No	221 (85%)
**Smoking history**	
Yes	83 (31.9%)
**Chronic medical history**	119 (45.8%)
Cardiovascular disease	56 (21.5%)
Cerebrovascular disease	42 (16.2%)
Digestive system disease	38 (14.6%)
Diabetes	30 (11.5%)
Chronic renal insufficiency	19 (7.3%)
Respiratory system disease	14 (5.4%)
Nervous system disease	6 (2.3%)
Malignant tumor	5 (1.9%)
Rheumatism and connective tissue disease	3 (1.2%)
**First signs and symptoms**	
Fever	235 (90.4%)
Cough	216 (83.1%)
Dyspnoea	181 (69.6%)
Myalgia	109 (41.9%)
Sputum production	71 (27.3%)
Generalized weakness	32 (12.3%)
Headache	23 (8.8%)
Chest pain	11 (4.2%)
Diarrhea	9 (3.5%)
More than one sign or symptom	248 (95.4%)
**CT findings**	
Unilateral pneumonia	43 (16.5%)
Bilateral pneumonia	217 (83.5%)
Multiple mottling and ground-glass opacity	31 (11.9%)

The median time from initial onset of symptoms to admission was 8 days (IQR 6.0–11.0; mean 7.8 ± 1.1). For initial symptoms, 235 (90.4%) patients had fever, 216 (83.1%) had cough, 181 (69.6%) had dyspnea, 109 (41.9%) had myalgia, and 71 (27.3%) had sputum production. Other symptoms included generalized weakness in 32 (12.3%) patients, headache in 23 (8.8%) patients, chest pain in 11 (4.2%) patients and diarrhea in 9 (3.5%) patients. Overall, 248 (95.4%) patients had one or more symptoms (Table 1).

On admission, chest CT abnormalities were detected among all patients, of which 217 (83.5%) showed bilateral pneumonia, 43 (16.5%) showed unilateral pneumonia, and 31 (11.9%) showed multiple mottling and ground-glass opacity. The typical findings of chest CT were bilateral multiple lobular and subsegmental areas of consolidation. Patchy lesions or multifocal peripheral ground-glass changes progressed to diffuse lesions usually within 4–6 days (Table 1).

On admission, the blood counts showed lymphopenia (< 1.1 × 10^9^/L) in 165 (63.5%) patients, with leukopenia (< 3.5 × 10^9^/L) in 20 (7.7%) patients. Leukocytes were above the normal range (> 9.5 × 10^9^/L) in 87 (33.5%) patients, and neutrophils were above the normal range (> 6.3 × 10^9^/L) in 103 (39.6%) patients. Seventy-three (28.1%) patients had decreased haemoglobin levels (< 130 g/L), and 47 (18.1%) patients had decreased serum platelets (< 125 × 10^9^/L). D-dimer levels were increased in 106 (40.8%) patients (Table 2).

**Table 2:** Laboratory findings of patients infected with COVID-2019 on admission.

	Patients (n = 260)
**Blood routine**	
Lymphocytes (× 10^9^ per L; normal range 1.1–3.2)	0.78 (0.45)
Decreased	165 (63.5%)
Leucocytes (× 10^9^ per L; normal range 3.5–9.5)	8.3 (3.2)
Increased	87 (33.5%)
Decreased	20 (7.7%)
Neutrophils (× 10^9^ per L; normal range 1.8–6.3)	5.3 (2.9–8.1)
Increased	103 (39.6%)
Decreased	15 (5.8%)
Platelets (× 10^9^ per L; normal range 125.0–350.0)	197.3 (84.5)
Increased	12 (4.6%)
Decreased	47 (18.1%)
Haemoglobin (g per /L; normal range 130.0–175.0)	121.6 (18.2)
Decreased	73 (28.1%)
D-dimer (μg per L;normal range 0.0–1.5)	0.86 (0.43–3.12)
Increased	106 (40.8%)
**Blood biochemistry**	
Albumin (g/L; normal range 35.0–52.0)	28.2 (7.4)
Decreased	223 (85.8%)
Alanine aminotransferase (U/L; normal range 0.0–41.0)	30.8 (17.0–48.0)
Increased	38 (14.6%)
Aspartate aminotransferase (U/L; normal range 0.0–40.0)	29.4 (17–45.0)
Increased	35 (13.5%)
Total bilirubin (μmol/L; normal range 0.0–26.0)	12.6 (8.2)
Increased	21 (8.1%)
Blood urea nitrogen (mmol/L; normal range 3.1–8.0)	5.5 (3.7)
Increased	19 (7.3%)
Serum creatinine (μmol/L; normal range 59.0–104.0)	77.2 (36.2)
Increased	12 (4.6%)
Creatine kinase (U/L; normal range 0.0–190·0)	99.2 (7.0–128.3)
Increased	51 (19.6%)
Decreased	40 (15.4%)
Lactate dehydrogenase (U/L; normal range 135.0–225.0)	298.0 (209.0–462)
Increased	155 (59.6%)
Troponin (pg/mL; normal range 0.0–34.2)	29.2 (8.2–47.3)
Increased	29 (11.2%)
Glucose (mmol/L; normal range 4.11–6.05)	7.8 (3.9)
Increased	105 (40.4%)
Decreased	8 (3.1%)
**Lymphocyte subpopulation**	
Decreased CD3+ (normal range 0.64–0.77)	68 (26.2%)
Decreased CD4+ (normal range 0.41–0.51)	97 (37.3%)
Increased CD8+ (normal range 0.23–0.33)	59 (22.7%)
Decreased CD4+/CD8+ (normal range 1.00–2.87)	106 (40.8%)
**Infection-related biomarkers**	
Erythrocyte sedimentation rate (mm per h; normal range 0.0–15.0)	43.2 (11.4)
Increased	198 (76.2%)
Interleukin-2 receptor (U/L; normal range 223–710)	700.8 (281–1278)
Increased	156 (60%)
Interleukin-6 (pg/mL; normal range 0.0–7.0)	8.4 (5.3–12.2)
Increased	149 (57.3%)
Interleukin-10 (pg/mL; normal range 0.0–9.1)	8.9 (6.2–13.0)
Increased	135 (51.9%)
Procalcitonin (ng/mL; normal range 0.0–0.05)	1.0 (1.7)
Increased	24 (9.2%)
Serum ferritin (ng/mL; normal range 30.0–400.0)	921.8 (511.6)
Increased	180 (69.2%)
C-reactive protein (mg/L; normal range 0.0–1.0)	47.5 (42.9)
Increased	192 (73.8%)
Tumor necrosis factor-α (pg/mL; normal range 0.0–8.1)	8.8 (4.9–13.6)
Increased	108 (40.8%)
**Co-infection**	
Other viruses	
Influenza A type IgM antibody	3 (1.2%)
Bacteria	17 (6.5%)
Mycoplasma pneumoniae IgM antibody	2 (0.8%)

On admission, 59 (22.7%) patients had differing degrees of liver function abnormality, with alanine aminotransferase (ALT) or aspartate aminotransferase (AST) above the normal range (Table 2). Patients had abnormal myocardial zymogram, which showed elevated creatine kinase (CK) in 40 (15.4%) patients and elevated lactate dehydrogenase (LDH) in 155 (59.6%) patients. Hypersensitive troponin I (hs-cTnI) was above the normal range in 29 (11.2%) patients, with whom the diagnosis of virus-related cardiac injury was associated. Thirty-one (11.9%) patients had different degrees of acute renal function damage, with elevated blood urea nitrogen or serum creatinine. Regarding the infection index, most patients had erythrocyte sedimentation rate (ESR), C-reactive protein (CRP), serum ferritin (SF) and interleukin-6 (IL-6) above the normal range (Table 2). Interleukin-2 receptor (IL-2R), interleukin-10 (IL-10), tumor necrosis factor-α (TNF-α) and procalcitonin (PCT) were above the normal range in 149 (57.3%), 135 (51.9%), 108 (40.8%), and 24 (9.2%) patients, respectively (Table 2). Many patients showed the decreased CD3+, CD8+, CD4+/CD8+ and increased CD8+ lymphocyte subpopulations (Table 2).

Bacteria and fungi were cultured from patients clinically suspected of having community-acquired pneumonia. Concomitant bacterial infections were detected in 27 (10.4%) patients, in whom most were older or had chronic underlying diseases. *Acinetobacter baumannii* was cultured in ten patients and *Klebsiella pneumoniae* in seventeen patients. *Acinetobacter* and Klebsiella turned out to be highly resistant to antibiotics. We found Influenza A type IgM antibodies in 3 (1.2%) patients and mycoplasma pneumoniae IgM antibodyiesin 2 (0.8%) patients. No fungi coinfections were detected (Table 2).

On admission, 209 (80.4%) patients presented with respiratory distress and respiratory rate above 30 times per minute. Mean oxygen saturation in the resting state was below 93% in 185 (71.2%) patients, and 79 (30.4%) patients had partial pressure of arterial oxygen to fraction of inspired oxygen ratios (PaO_2_/FiO_2_) below 300 mmHg. During hospitalization, lung infiltrate progression was above 50% within 24–48 h in 16 (6.2%) patients (Table 3).

**Table 3:** Clinical management of patients with COVID-2019.

Duration from admission to discharge	All patients (N = 260)
**Definitions of severe patients**	
Respiratory rate > 30/min	209 (80.4%)
Mean oxygen saturation < 93%	185 (71.2%)
PaO_2_/FiO_2_ ≤ 300 mmHg	79 (30.4%)
Lung lesion progression > 50% within 24-48 h	16 (6.2%)
**Treatment**	
Antiviral therapy	260 (100%)
Antibiotic therapy	260 (100%)
Use of corticosteroid	103 (39.6%)
**Oxygen support**	
Nasal catheter	176 (67.7%)
Mask oxygen	84 (32.3%)
High-flow nasal cannula	113 (43.5%)
**Assisting ventilation**	
Bipap	47 (18.1%)
Invasive mechanical ventilation	12 (4.6%)
**Outcome**	
Discharge	183 (70.4%)
Continue to be hospitalized	35 (13.5%)
ICU	17 (6.5%)
Death	25 (9.6%)

Each patient received 4–5 therapies, including nutrition support, supplemental oxygen, antiviral treatment, antibiotic and/or methylprednisolone (39.6% patients). All patients received treatment criteria according to the Chinese management guideline for COVID-19 (version 7.0)^8^. Individualized treatment of patients and selection of therapies were guided by presenting illness severity and were not arbitrary. Effective oxygen therapy was given by nasal catheter, mask oxygen and transnasal high-flow oxygen when saturations as measured by pulse oximeter dropped below 93% (Table 3). All patients received antiviral treatment with interferon alpha inhalation (50 μg twice daily), lopinavir and ritonavir (500 mg twice daily, orally), Riba Welin (500 mg twice daily, intravenously), arbidol (200 mg twice daily, orally), and chloroquine phosphate (500 mg twice daily, orally). Each patient took one or two antiviral drugs, and the median antiviral treatment length was 7 days (IQR 3–7) (Table 2).

Overall, 103 (39.6%) patients received treatment with methylprednisolone (1–2 mg/kg/day) for a median of 12 days (IQR 7–15) and gamma globulin (15–20 g/day) for a median of 10 days (IQR 6–12) when severe patients had a significant increase in the amounts of pro-inflammatory cytokines, such as IL-2R, IL-6, IL-10, CRP, TNF-α, and IFN-γ, which was characterized as a cytokine storm. Once the patient was found to have a cytokine storm and systemic inflammatory response syndrome, or multiple pulmonary lobes showed more than 50% progression in 48 h on chest CT. Meanwhile, patients received chest CT reexaminations based upon changes in clinical status. None of the patients used hormone shock therapy. All patients were administered empirical antibiotic treatment (oral or intravenous). The antibiotics used generally covered common pathogens and some atypical pathogens; when secondary bacterial infections occurred, medication was administered according to the results of bacterial culture and drug sensitivity. The antibiotics used were cephalosporins (Sulperazone), quinolones, carbapenems, tigecycline against methicillin-resistant *Staphylococcus aureus* and linezolid. Overall, 107 (41.2%) patients were treated with a single antibiotic, and 153 (58.8%) patients were given combination therapy. The median of antibiotic treatment duration was 5 days (IQR 3–7). Patients also received treatment with probiotics for cases of gastrointestinal symptoms (Table 2).

Invasive mechanical ventilation was required in 12 (4.6%) patients, with a median of 7.5 days (IQR 6–17). Forty-seven (18.1%) patients used a bipap ventilator for a median of 5 days (IQR 4–14). Four patients used an invasive ventilator to assist with ventilation, with a median 7 days (IQR 6–12). The ventilator adopted PSIMV mode, the inhaled oxygen concentration was 30–100%, and the positive end expiratory pressure was 6–12 cm H_2_O. Ten patients were still using ventilator assisted breathing at the data cutoff. Moreover, 8 (3.1%) patients with acute kidney injury received continuous blood purification due to renal failure (Table 3).

There were no significant differences based upon treatment strategy or clinical outcome. No patients with only radiographic progression without concomitant clinical deterioration were observed. The trajectory of laboratory data for each patient was dynamically monitored during treatment. Laboratory data between admission, prognosis and outcome of disease are shown in Table 4. Average plasma lymphocyte, D-dimer, CD3+ T cell, CD4+ T cell, CD8+ T cell, ESR, IL2R, IL6, IL10, PCT, serum ferritin and TNFα levels recovered to normal with statistical significance when cured patients were compared to those at admission. However, these indicators showed aggravation or deterioration to death (Table 4).

**Table 4:** Laboratory data between admission, prognosis and outcome of disease.

	All patients (n = 260)	Cured (p value) (n = 183)	Death (p value) (n = 25)	Continued hospital (p value) (n = 52)
**Blood routine (unit; normal range)**				
Lymphocytes (× 10^9^ per L; 1.1–3.2)	1.0 ± 0.76	1.9 ± 1.3 (0.02)	0.81 ± 0.12 (< 0.001)	1.02 ± 0.76 (0.57)
Leukocytes (× 10^9^ per L; 3.5–9.5)	7.20 ± 2.1	7.15 ± 3.1 (0.13)	7.23 ± 3.7 (0.11)	7.3 ± 3.7 (0.079)
Neutrophils (× 10^9^ per L; 1.8–6.3)	6.30 ± 1.8	6.21 ± 1.5 (0.10)	6.41 ± 3.2 (0.01)	6.24 ± 1.5 (0.27)
D-dimer (unit; normal range) (μg per L; 0.0–1.5)	1.86 ± 5.9	0.72 ± 1.7 (< 0.001)	2.72 ± 3.7 (< 0.001)	1.92 ± 4.9(0.013)
**Lymphocyte subpopulation (normal range)**				
CD3+ T cell (0.64—0.77)	0.61 ± 0.18	0.70 ± 0.27 (0.007)	0.53 ± 0.46 (< 0.001)	0.62 ± 0.34 (0.063)
CD4+ T cell (0.41–0.51)	0.40 ± 0.13	0.44 ± 0.26 (0.012)	0.37 ± 0.19 (< 0.001)	0.41 ± 0.16 (0.071)
CD8+ T cell (0.23–0.33)	0.33 ± 0.13	0.32 ± 0.09 (0.08)	0.34 ± 0.10 (0.086)	0.33 ± 0.23 (0.19)
CD4+/CD8+ (1.00–2.87)	1.66 ± 1.21	2.0 ± 1.03 (< 0.001)	0.91 ± 3.03 (< 0.001)	1.60 ± 2.34(0.007)
**Infection-related biomarkers (unit; normal range)**				
Erythrocyte sedimentation rate (mm per h; 0.0–15.0)	43.2 ± 17.6	7.2 ± 9.6 (< 0.001)	36.8 ± 13.4 (0.053)	36.8 ± 13.4 (< 0.001)
Interleukin-2 receptor (U/L; 223–710)	700.8 ± 347.6	521.6 ± 223.2(< 0.001)	820.4 ± 447.6(< 0.001)	716.4 ± 308.1 (0.001)
Interleukin-6 (pg/mL; 0.0–7.0)	8.4 ± 12.2	5.0 ± 13.7 (< 0.001)	10.1 ± 27.0 (< 0.001)	8.3 ± 17.1 (0.09)
Interleukin-10 (pg/mL; 0.0–9.1)	8.9 ± 7.9	4.7 ± 6.2 (< 0.001)	9.78 ± 21.34 (< 0.001)	8.68 ± 8.34(0.041)
Procalcitonin (ng/mL; 0.0–0.05)	1.0 ± 1.4	0.03 ± 1.7 (0.005)	1.1 ± 2.7 (0.039)	1.0 ± 2.7 (0.086)
Serum ferritin (ng/mL; 30.0–400.0)	921.8 ± 511.6	321.8 ± 111.6 (< 0.001)	900.8 ± 601.1(< 0.001)	477.6 ± 212.6 (< 0.001)
C-reactive protein (mg/L; 0.0–1.0)	47.5 ± 42.9	0.75 ± 0.09 (< 0.001)	78.2 ± 36.8 (< 0.001)	10.2 ± 8.6 (< 0.001)
Tumor necrosis factor-α (pg/mL; 0.0–8.1)	8.8 ± 13.6	4.1 ± 3.7 (< 0.001)	11.2 ± 9.4(< 0.001)	8.0 ± 11.6 (0.116)

At the data cutoff for this study, on March 10, 2020, 183 (70.4%) patients had been cured and discharged, and the median time from hospital admission to discharge was 21 days (IQR 10–36; mean 21.8 ± 7). Seventeen (6.5%) patients were transferred to the ICU due to respiratory failure, septic shock, and/or multiple organ dysfunction, and 25 (9.6%) patients had died. The twenty-five deaths were elderly patients (median age of 61 years, IQR 58–82; mean 66.9), and most had chronic underlying disease. One 62-year-old male patient died of myocarditis with abnormal hs-cTnI (410 pg/mL), CK (3160 U/L) and LDH (6120 U/L). The deaths were due to severe respiratory failure, heart failure, myocarditis, septic shock and multiple organ dysfunction. Of these, lung lesions on chest CT progressed rapidly in ten patients in a short period (4 days, IQR 3–6). Of the remaining 35 (13.5%) patients were still in the hospital (Table 3).

Patients who were cured and discharged were required to go to a designated place for unified implementation of rehabilitation isolation and medical observation for 14 days. During the follow-up period, 7 patients who had fever (> 37.3 °C), and were retested for SARS-Cov-2 antigens were negative.

## Discussion

The clinical features and outcomes of patients with severe COVID-2019 are very limited, and there is still a lack of standardized management guidelines and research on severe patients, and need to be further explored. This observational study presented the latest status of patients with severe SARS-CoV-2 infection in Wuhan China and added a short-term follow-up. Overall, 260 patients with severe COVID-19 were included in this study.

The median age of severe patients was 61 years old, and the predisposing conditions for COVID19 pneumonia tended to be elderly and those with chronic medical conditions (such as cardiovascular and cerebrovascular disease diabetes, digestive system disease, diabetes and other chronic diseases), similar to previous viral infections, such as influenza A^9,10^. There was no obvious predilection for males or females in our cohort. Most (85%) patients had no known history of exposure to the market, but all lived in Wuhan for 14 days before the onset of illness, they could have acquired the infection from the community or the hospital. The proportions of patient with family cluster and medical staff was less. Transmission rates were unknown for COVID-19^6^. Nevertheless, these findings suggested that human-to-human transmission and intercity spread of COVID-19 were conceivable.

The median time from initial onset of symptoms to admission was 8 days (IQR 6.0–11.0; mean 7.8 ± 1.1), which was longer than that previously reported in all patient classifications^6^. Common clinical presentations at onset of illness greatly resembled SARS-CoV, with fever, dry cough, dyspnoea, myalgia, sputum, generalized weakness, headache and chest pain^11–13^. COVID-19 patients rarely developed diarrhea or nausea. Most patients presented initially with one or more signs or symptoms. A significant proportion of patients had atypical symptoms, such as generalized weakness and headache. Few patients with COVID-19 had prominent upper respiratory tract signs and symptoms (e.g., rhinorrhoea, sneezing, or sore throat), indicating that the target cells might be located in the lower airway, similar to previous reports^11–16^. Bilateral distribution of multiple mottling shadows and ground-glass opacity were typical hallmarks of chest CT for COVID-19. For patients who were cured and discharged, substantially improved acute exudative lesions were observed in chest CT images.

The most common laboratory abnormalities observed in this study were decreased albumin, lymphopenia, increased d-dimer, and elevated lactate dehydrogenase. Elevated levels of high-sensitivity cardiac troponin I, creatine kinase, alanine and aspartate aminotransferase, blood urea nitrogen, and serum creatinine could be detected in many patients. Decreased albumin suggested the importance of nutritional support. These abnormalities suggested that SARS-CoV-2 infection may be associated with coagulation activation, myocardial injury, hepatic injury, acute kidney injury and cellular immune deficiency. The decrease in the number of T cell subpopulations and lymphocytopenia suggested that SARS-CoV-2 might mainly act on lymphocytes, especially T lymphocytes, as observed in previous betacoronavirus infections^14,15^. These laboratory abnormalities were similar to those previously observed in patients with Middle East Respiratory Syndrome-related coronavirus (MERS-CoV) and Severe acute respiratory syndrome coronavirus (SARS-CoV) infection^14–16^.

Elevated levels of infection-related biomarkers (ESR, IL2R, IL6, IP10, PCT, SF, CRP, TNFα) were detected in many patients with severe COVID-19. Moreover, 12 patients transferring to the ICU had higher concentrations of infection-related biomarkers (ESR, IL2R, IL6, IP10, PCT, SF, CRP, TNFα). The significantly increased concentrations of cytokines may be associated with a cytokine storm induced by virus invasion. Virus particles spread through the respiratory mucosa and infect other cells, induce a cytokine storm in the body, generate a series of immune responses, and cause changes in peripheral white blood cells and immune cells, such as lymphocytes^17,18^. The dynamic profile of laboratory values was tracked in all patients. When the general conditions of patients improved, the values of infection biomarkers, especially IL-2R, IL-6 and CRP, significantly decreased. The findings indicated that IL-2R, IL-6 and CRP have certain advantages in predicting the severity of illness. Therefore, we speculate that IL-2R, IL-6 and CRP are associated with the severity of COVID-19. Moreover, many patients showed decreased CD3+, CD8+, CD4+/CD8+ and increased CD8+ lymphocyte subpopulations. Some studies have suggested that a substantial decrease in the total number of lymphocytes indicates that coronavirus consumes many immune cells and inhibits the body’s cellular immune function^14,15^. Damage to T lymphocytes and cytokine storms might be important factors leading to exacerbations of patients. The low absolute value of lymphocytes could be used as a reference index in the diagnosis of SARS-CoV-2 infection. Therefore, the early identification and timely treatment of severe cases is of crucial importance. Intravenous immunoglobulin was recommended to enhance the treatment for severe patients, and steroids (methylprednisolone 1–2 mg/kg per day) were recommended to weaken cytokine storms, for as short a duration of treatment as possible. However, previous studies in patients with SARS and MERS suggested that receiving corticosteroids did not have an effect on mortality, but rather delayed viral clearance^16,17^. WHO, based on a prospective meta-analysis of randomized clinical trials, recommended the use of systemic glucocorticoids for the treatment of patients with severe and critical COVID-19^18^. Recent cohort study showed that corticosteroid treatment was associated with beneficial outcomes in a subset of COVID-19 patients who were non-diabetic and with severe symptoms^19^. Whether the use of steroids affects the prognosis of patients remained unknown^20^. We suppose that steroids might be a beneficial part of the treatment for patients with severe COVID-19 but should be used with caution.

Until now, no specific antiviral treatment has been recommended for coronavirus infection except for meticulous supportive care^17,21,22^. The combination of antiviral treatment (interferon alpha, lopinavir and ritonavir, Riba Welin, arbidol, chloroquine phosphate, less than two antiviral drugs per patient) among SARS-CoV patients was associated with substantial clinical benefit (fewer adverse clinical outcomes, 3–10 days) in this study. Treatment with lopinavir and ritonavir were reported to have the potential to treat MERS/SARS infections^22,23^. Therefore, we suppose this might be a beneficial part of the treatment for COVID-19. The length of antiviral treatment should be cautiously considered. However, the types of drugs used varied among patients. Previous study showed early triple antiviral therapy was safe and superior to lopinavir-ritonavir alone in alleviating symptoms and shortening the duration of viral shedding and hospital stay^23^. The use of lopinavir/ritonavir and ribavirin alone is not recommended in patients with COVID-19 and hydroxychloroquine is not recommended^24^.

Some patients, who tended to be older and had underlying chronic disease, had bacterial coinfections. For severe mixed infections, in addition to the virulence factors of pathogens, the host’s immune status was also an important factor. Old age and the presence of comorbidity might be associated with increased mortality. When populations with low immune function, such as the elderly, chronic renal insufficiency, diabetics, people with long-term use of immunosuppressive agents, and pregnant women, were infected with SARS-CoV-2, prompt administration of antibiotics to prevent infection and strengthening of immune support treatment might reduce complications and mortality. All of the patients in this study received antibacterial agents and were administered with empirical antibiotic treatment and the results of bacterial culture and drug sensitivity. Although no direct effective outcomes were observed, serum blood cell count, IL2R, IL6 and CRP in this study showed decreases or returns to normal range. Effective antibacterial treatment might improve outcomes in COVID-19. When severe patients develop to ARDS, mechanical ventilation is the main supportive treatment. Airway management should be considered in severe and critical cases with COVID-19^24^. In this cohort, 12 (4.6%) patients required invasive mechanical ventilation, and 47(18.1%) patients used a noninvasive bipap ventilator.

At the data cutoff for this study, on March 10, 2020, 17 (6.5%) patients were transferred to ICU due to respiratory failure, septic shock, and/or multiple organ dysfunction. Overall, 25 (9.6%) patients died, most of whom were old, or had underlying comorbidities, mainly cardiovascular and cerebrovascular diseases and diabetes, resembling MERSCoV. As of March 10, 2020, the overall case-fatality rate (CFR) in Wuhan was 2423 (4.8%) of 49,978 confirmed cases with COVID-19 from the national official statistics. In total 183 (70.4%) patients had been cured and discharged, and the mean interval from admission to discharge was 22.0 days, with longer hospital stays. Of the remaining, 35 (13.5%) patients are being treated in isolation at the hospital. However, additional deaths might occur in those still hospitalized. The CFR was 61.5% among critical cases. The CFR of severe patients in our cohort was higher than the overall CFR but was much lower than the CFR of critical cases. Since additional deaths might occur in those still hospitalized, the actual CFR might be even higher than the present rate. Nevertheless, SARS-CoV-2 appears to have a lower case fatality rate than either SARS-CoV or MERS-CoV^25,26^. These findings suggest that the cure rate for severe cases with SARS-CoV-2 infection is quite high and the CFR relatively lower. Centralized isolation management and stratification might collectively contribute to boosting the cure rate. During the period of follow-up, SARS-Cov-2 antigen retesting was negative in seven patients with fever. Even so, we still recommend a designated place for unified implementation of rehabilitation isolationfor a period of 14 days and plus medical observation for 14 days^24^.

This study has several limitations. First, this study did not include critical cases or generally mild cases. There may also be a selection bias when identifying the clinical outcomes. The true CFR of all classification COVID-19 should be lower. Second, the described study did not set a control group and was not driven by formal hypotheses. Further, some randomly controlled studies are still needed. Third, our study was limited to patients from a single isolation district, which may limit its generalizability to other hospitals. However, our hospital was the designated hospital with the most severe and critical patients, and we believe our study population was representative of cases diagnosed and treated in Wuhan. A larger cohort study of patients with COVID-19 pneumonia from other hospitals would help to further define the clinical characteristics and outcomes.

In conclusion, the cure rate for patients with severe COVID-19 was actually quite high, and the CFR was relatively lower. Severe patients might be associated with the elderly, medical comorbidities, longer waiting time to hospitalization, and cytokine storm, especially elevated levels IL2R, IL6 and CRP. SARS-CoV-2 may consume many immune cells and inhibit the body’s cellular immune function. The deaths mainly occurred in patients who were older and had medical comorbidities. However, SARS-CoV-2 is highly infectious and could be a significant health threat. For patients with COVID-19 pneumonia, centralized isolation management, stratified therapy and early treatment might collectively contribute to boosting cure rates and controlling disease transmission. Longer follow-ups of severe patients are necessary to observe long-term outcomes.

## Methods

### Study design and participants

In this prospective study, we included cases with severe COVID-19 admission from Jan 19 to Feb 19, 2020, in an isolation ward of Wuhan Tongji Hospital, which was designated to receive and treat severe and critical patients. Case definitions of confirmed human infection with SARS-Cov-2 were in accordance with the interim guidance from the WHO. Severe cases were defined as any of the following: respiratory distress and respiratory frequency above 30 times per minute; mean blood oxygen saturation in the resting state below 93%; partial pressure of arterial oxygen (PaO_2_) to fraction of inspired oxygen ratio (FiO_2_) below 300 mmHg; and lung infiltrates above 50% within 24–48 h. Only patients with a laboratory confirmed infection were enrolled in this study. All methods were performed in accordance with the Chinese management guideline for COVID-19 (version 7.0)^8^. This study was approved by Wuhan Tongji Hospital ethics committee, and the requirement for informed consent was waived by the Ethics Commission due to the observational nature of the study^27^.

### Laboratory procedure

All laboratory procedures for clinical samples had been previously reported^4,28–30^. Briefly, throat-swab specimens from the upper respiratory tract were obtained on admission and put into viral transport medium. SARS-CoV-2 was confirmed by real-time polymerase chain reaction (RT-PCR) within three hours. RT-PCR detection reagents were provided by Wuhan Tongji Hospital, which is a certified tertiary care hospital. Throat-swab specimens were obtained for SARS-CoV-2 PCR re-examination every other day after clinical remission of symptoms, including fever, cough, and dyspnea, but only qualitative data were available. RT-PCR assays were performed in accordance with the protocol established by the WHO^3^. All patients were tested for nine respiratory pathogens and the nucleic acid of influenza viruses A and B. Sputum or endotracheal aspirates were obtained for the identification of possible causative bacteria or fungi. Plasma was separated from EDTA bottles. Laboratory tests were conducted on admission, including a complete blood count, serum biochemistry, coagulation profile, infection-related biomarkers, myocardial enzymes, myoglobin, troponin, and T cell subpopulations. All laboratory tests were performed according to the manufacturer’s instructions.

### Clinical management

All patients received treatment according to the Chinese management guideline for COVID-19 (version 7.0)^8^. Laboratory tests and chest CT scans were re-examined upon clinical remission of symptoms. We recorded data on age, sex, exposure history, chronic medical histories, symptoms from onset to hospital admission, vital signs on admission and hospitalization, laboratory values, coexisting infection, treatment, as well as living status on electronic medical records.

All the following criteria had to be met for hospital discharge^8^: significant improvement in respiratory symptoms, normal temperature lasting longer than 7 days, substantially improved acute exudative lesions in chest CT, and three consecutive throat-swab samples negative for SARS-CoV-2 RNA at least 24 h apart. The discharged patients were isolated for 14 days in a fixed accommodation arranged by the government. These patients were followed up to Mar 25, 2020 by telephone lasting more than two weeks.

### Data collection

At the data cutoff for this study, on March 10, 2020, we recorded and evaluated clinical charts, nursing records, laboratory findings, and chest CT for all included cases. The data on epidemiological, clinical, laboratory, chest CT, treatment and outcomes were acquired from electronic medical records with a standardized data collection form (modified version for severe acute respiratory infection clinical characterization shared by the International Severe Acute Respiratory and Emerging Infection Consortium). Two trained researchers independently reviewed the data collection forms to double check the data collected.

### Statistical analysis

Descriptive analyses of the variables were expressed as the medians (interquartile range, IQR) or numbers (%). We presented continuous measurements as the means (SD) if they were normally distributed or medians (IQR) if they were not and categorical variables as counts (%). For laboratory values, we also assessed whether the measurements were outside the normal range. SPSS (version 26.0) was used for all analyses.
